# Rater characteristics, response content, and scoring contexts: Decomposing the determinates of scoring accuracy

**DOI:** 10.3389/fpsyg.2022.937097

**Published:** 2022-08-10

**Authors:** Corey Palermo

**Affiliations:** Measurement Incorporated, Durham, NC, United States

**Keywords:** rater effects, writing assessment, rater-mediated assessment, multilevel modeling, rater monitoring

## Abstract

Raters may introduce construct-irrelevant variance when evaluating written responses to performance assessments, threatening the validity of students’ scores. Numerous factors in the rating process, including the content of students’ responses, the characteristics of raters, and the context in which the scoring occurs, are thought to influence the quality of raters’ scores. Despite considerable study of rater effects, little research has examined the relative impacts of the factors that influence rater accuracy. In practice, such integrated examinations are needed to afford evidence-based decisions of rater selection, training, and feedback. This study provides the first naturalistic, integrated examination of rater accuracy in a large-scale assessment program. Leveraging rater monitoring data from an English language arts (ELA) summative assessment program, I specified cross-classified, multilevel models via Bayesian (i.e., Markov chain Monte Carlo) estimation to decompose the impact of response content, rater characteristics, and scoring contexts on rater accuracy. Results showed relatively little variation in accuracy attributable to teams, items, and raters. Raters did not collectively exhibit differential accuracy over time, though there was significant variation in individual rater’s scoring accuracy from response to response and day to day. I found considerable variation in accuracy across responses, which was in part explained by text features and other measures of response content that influenced scoring difficulty. Some text features differentially influenced the difficulty of scoring research and writing content. Multiple measures of raters’ qualification performance predicted their scoring accuracy, but general rater background characteristics including experience and education did not. Site-based and remote raters demonstrated comparable accuracy, while evening-shift raters were slightly less accurate, on average, than day-shift raters. This naturalistic, integrated examination of rater accuracy extends previous research and provides implications for rater recruitment, training, monitoring, and feedback to improve human evaluation of written responses.

## Introduction

Large-scale summative assessments typically include open-ended items requiring students to construct written responses. Scoring these constructed responses involves recruiting, training, certifying, and monitoring hundreds or (in the case of the present study) even thousands of raters. Raters are trained to apply scoring criteria—in particular rubrics and anchor responses—to score student responses accurately. However, raters do not always apply the scoring criteria appropriately and may assign a higher or lower score than warranted to a response. Such errors in judgment, known as rater effects, introduce construct-irrelevant variance and threaten the validity of student scores. Rater effects can compromise the fairness of an assessment by impacting student achievement estimates and classification decisions (Wind, 2019).

The *Standards for Educational and Psychological Testing* (AERA et al., 2014) consequently call for monitoring human scoring quality and correcting any systematic scoring errors identified in an assessment program, as part of ensuring the assessment demonstrates acceptable psychometric quality. Despite the persistence of rater effects, more needs to be learned about the sources of inter- and intra-rater discrepancies in order to efficiently and effectively attend to them in practice. Numerous factors in the rating process, including the content of students’ responses, characteristics of raters, and the context in which the scoring occurs, are thought to influence the quality of raters’ scores.

### Response content

Response content can comprise the text features of a response (e.g., lexical characteristics), the information or ideas included in the response (e.g., semantic characteristics), and even the visual appearance of the response (e.g., legibility, formatting). Few studies have examined how the content of students’ responses to test items impact rater accuracy. Recent research in this area has been conducted in the context of language testing programs and is thus beyond the scope of this review (e.g., Weigle et al., 2003; Schoonen, 2005; Barkaoui, 2010). In the context of a large-scale, ELA assessment, Leacock and colleagues (Leacock et al., 2013; Leacock and Zhang, 2014) identified several characteristics of responses and associated items that affected rater agreement. They found lower rater agreement associated with longer responses and items that were more cognitively complex, required inferencing, and offered numerous text-based key elements to draw from. Wolfe et al. (2016) examined text features of difficult-to-score essays in the context of a direct writing assessment. The authors found the length and lexical diversity of responses to explain 25% of the variance in ease of scoring. Shorter and more lexically diverse essays were easier for raters to score accurately.

Several studies that have used cross-classified, multilevel analysis to evaluate rater accuracy provide further evidence that the content of students’ responses can influence rater accuracy (e.g., Baird et al., 2013, 2017). Multilevel modeling approaches allow for partitioning sources of variance in rater accuracy, and researchers have found, after controlling for rater and team effects, individual responses—and associated items—to be a significant source of variance in rater accuracy. Baird et al. (2013) analyzed scoring data associated with England’s AS-level exams and found significant effects of items and responses in the areas of geography and psychology. Using scoring data from England’s A-level examinations, Baird et al. (2017) found responses to account for up to 2% of the variance in rater accuracy. Items explained an additional 4–7% variance in rater accuracy, depending on the subject area. Collectively, these findings suggest that response content can influence the quality of raters’ evaluations.

### Rater characteristics

Rater characteristics are personal and psychological attributes of raters, including experience, credentials, demographics, and personality traits. Much of the extant research examined the association between rater characteristics and rating quality in the context of language testing programs (see Song et al., 2014, for a review). A limited number of studies conducted in the United Kingdom have investigated the impact of rater characteristics on scoring accuracy and consistency in summative assessment programs. Researchers have explored the scoring accuracy of raters with differing scoring experience, subject knowledge, and teaching experience in the context of England’s national examinations. Raters demonstrated comparable accuracy scoring English assessments despite disparate backgrounds (Royal-Dawson and Baird, 2009; Meadows and Billington, 2010). Meadows and Billington (2010) found that experienced raters were slightly more consistent than raters who lacked subject knowledge and teaching experience. Leckie and Baird (2011) reported that novice raters, experienced raters, and team leaders exhibited similar overall accuracy and changes in accuracy over time when scoring an English essay. However, the authors found evidence of significant intra-rater variation in accuracy over time. Results of these studies suggest a tenuous relationship between rater characteristics and scoring accuracy.

In the United States, Song et al. (2014) examined the impact of rater demographics and experience on the scoring of ELA essay and science short answer responses. The authors examined predictors including demographics (ethnicity, gender, age) and credentials (degree and teaching experience). Rater performance was measured using validity and inter-rater reliability. The authors did not find evidence that rater background was associated with score quality.

Little attention has been given to rater qualification performance, despite the high-stakes nature of rater qualification assessments in ensuring rater employment and, at least theoretically, score quality. Investigations of rater qualification tests have found them to have generally low reliability and decision consistency, due in large part to length—often no more than two sets of 10 responses each (Ricker-Pedley, 2011; Attali, 2019). In short, much remains to be learned about the relations between rater qualification performance and operational scoring accuracy.

### Scoring contexts

Scoring contexts include the scoring organizational structure and the physical and/or virtual scoring environment, including scoring technologies employed, in addition to procedures for rater training and supervision. In large-scale summative assessment scoring each rater is assigned to a team with a supervisor for the purpose of training and monitoring. Limited previous research has examined team or supervisor effects on rating quality. Baird et al. (2017) reported training team effects on rater accuracy, with team variance constituting 8–16% of the variation in rater accuracy, depending on the type of monitoring system used. Baird et al. (2013) applied multiple analysis techniques and found differing results: a cross-classified multilevel model showed small team effects on raters’ scoring of open-ended geography items, while a generalizability study showed no evidence of team effects on the same items. While these empirical results provide some evidence of team effects on scoring accuracy, more comprehensive research is needed to better understand the impact of team membership within the greater context of rater effects.

Researchers have also examined the extent to which the training and scoring environment and process impacts raters’ score quality. This literature suggests that raters can be trained online as effectively—and likely more efficiently—than in person (Knoch et al., 2007, 2018). Evidence from a quasi-experimental study suggests that raters may demonstrate comparable accuracy and reliability when scoring in distributive and site-based settings (Wolfe et al., 2010). Rater perceptions of training and scoring effectiveness and satisfaction have been found to be consistent across scoring contexts (Wolfe et al., 2010; Knoch et al., 2018). Though remote training and scoring programs are increasingly prevalent, the field lacks ecologically valid investigations of rater effects in remote scoring contexts.

### Integrated approaches

In sum, a sparse but mounting literature base suggests that response content as well as a wide variety of components of the scoring context influence scoring accuracy. Rater characteristics are thought to have lesser influence on scoring accuracy. However, prior studies have largely examined these various sources of rater effects in isolation, leaving the relative impact of the factors thought to influence rater accuracy unresolved. Moreover, the academic nature of much of this work has limited its application in operational assessment programs.^1^

Suto et al. (2011) provide a rare example of an integrated approach to investigating rater accuracy. They examined the relative effects of a variety of factors, including raters’ experience, education, and rating task demands (e.g., cognitive complexity, score points) on scoring accuracy in the context of an International General Certificate in Secondary Education in biology examination. Their results showed differential relations between education (general and relevant) and accuracy for items that were more cognitively complex to evaluate. The association between education and accuracy was also moderated by qualification performance; however, the study’s experimental design deviated from operational marking in that it included raters who did not meet the qualifying criteria.

Suto et al. (2011) provided results showing preliminary evidence of interrelations among the factors that influence rater accuracy. They further highlight the importance of integrated examinations to afford evidence-based decisions of rater selection, training, and feedback. Failing to account for relevant determinates and correlates of rater effects risks biasing results of rater accuracy investigations. Only through understanding relative impacts of the factors that influence rater accuracy can scoring errors adequately be prevented and fair assessment practices ensured.

### The present study

The present study aims to overcome two primary limitations of the reviewed research. First, previous results have been subject to considerable limitations including study scope (e.g., Leckie and Baird, 2011; Wolfe et al., 2016), sample size (e.g., Suto et al., 2011; Song et al., 2014), and raters’ awareness of monitoring tools (e.g., Leckie and Baird, 2011; Baird et al., 2017). Second, many of the published findings have been the result of designed studies conducted in controlled, artificial settings (e.g., Royal-Dawson and Baird, 2009; Meadows and Billington, 2010; Wolfe et al., 2010), rather than real-world scoring contexts. These limitations carry threats to external and ecological validity, impeding representativeness and generalizability of findings.

The present study offers the first naturalistic, integrated examination of rater accuracy in the context of a large-scale assessment program. In the study, I decompose the impact of response content, rater characteristics, and scoring contexts on rater accuracy to gain insight into the relative impact of factors that influence rater accuracy and establish an evidence base with utility to improve practice and policy. I examine the determinates and correlates of rater accuracy using rater-monitoring data produced during the scoring of ELA summative assessments in the United States. Accuracy was evaluated by comparing raters’ scores to the benchmark scores of responses. Benchmark scores are the criterion or “true” scores determined to be most accurate by experts. These data are applied to address the following research questions:

1. To what extent did scoring accuracy vary across teams, items, raters, responses, and time?
2. To what extent did response content, rater characteristics, and scoring context influence scoring accuracy?

## Materials and methods

### Assessments

The ELA assessments were administered in Spring 2017 to students in grades 3–11. Approximately 1.7 million students across 11 states and territories in the United States completed the assessments online, in addition to a few thousand students who completed paper tests. Constructed response items assessed students’ writing, research, and reading skills. The writing items required that students write or revise a short text of one or more paragraphs and included multiple writing purposes (opinion/argumentative, narrative, informative/explanatory). The research items required that students investigate topics by analyzing, integrating, and presenting information. These items were scored on a 0–2 scale. I exclude the reading items from analysis, as these were exposed to a relatively small proportion of examinees and were scored by few raters. I also exclude the writing prompts (i.e., essays) from this investigation, to maintain focus on the scoring of shorter constructed responses.

### Scoring process: raters, training, and monitoring

Eligibility requirements for prospective raters included a 4-year degree, successful completion of an interview, and provision of references. Scoring team leaders were former raters with experience working on multiple scoring projects.

Training followed a standardized process whether raters worked in a scoring site or remotely. To achieve this consistency, the scoring directors who conducted training on-site created scripted videos used to deliver remote lessons. Raters were trained first on a scoring rubric specific to either each item (for the research items) or a writing purpose and grade band (for the writing items), and then on a set of anchor responses that exemplified each score point of the rubric. Raters next completed one or more practice sets of responses and received feedback on their performance. Finally, to qualify, raters were required to achieve 70% accuracy on at least one of two, 10-response qualification sets. Assigning a non-adjacent score automatically disqualified a rater from passing a given set. Raters typically trained and qualified to score several similar items.

Raters were assigned to teams so that during scoring team leaders could monitor raters’ performance. All scoring was conducted within a secure, online system. Here, raters accessed scoring sets comprising 5–10 student responses associated with a single item they had qualified to score. Raters reviewed each response, using a drop-down menu to assign the most appropriate score based on their knowledge of the rubric, anchor responses, and other insight gained during training. As a safeguard, the scoring system required that raters view each page of lengthy responses prior to entering a score. If a rater had a question about a given response they could transfer it, along with a digital note, to their team leader for direction. Prior to submitting the scoring set, raters had the opportunity to review all responses and assigned scores.

Within this system, validity responses (i.e., expert-scored benchmark responses) were interleaved with operational responses and thus distributed inconspicuously to raters. Rater scores could be compared to the expert scores of the validity responses to evaluate rater accuracy. Validity responses comprised 5% of all operational responses scored. Additionally, 15% of responses were scored independently by two raters to monitor inter-rater reliability (IRR). Based on validity and IRR results, supervisors conducted targeted read-behinds by auditing scores assigned by a particular rater to further assess performance, diagnose challenges, and provide feedback.

### Data

During operational scoring of the assessments, the raters assigned a total of 644,670 scores to validity responses. These scores spanned 15,087 unique validity responses, 98 teams, 432 items, and 1,329 raters. I calculated the absolute difference between the rater score and the expert-assigned score for each validity response to create the dependent variable, accuracy. The dependent variable measured the extent to which the rater-assigned scores deviated from the benchmark scores, with larger values indicating greater disagreement. This variable had a mean of 0.15, suggesting relatively accurate scoring, on average.^2^ However, a standard deviation of 0.38 indicated moderate variation about this average. A predictor variable for time, measured as the number of days since the rater’s first-assigned score,^3^ was created to examine change in accuracy over time. This variable had a mean of 15.93 (*SD* = 14.88).

#### Response content

Content of the validity responses was examined directly, via text features, and indirectly, via assessment expectations and grade bands to which items were aligned. Text feature variables were obtained using the automated scoring engine *Project Essay Grade* (PEG). A detailed description of PEG is beyond the scope of this paper; interested readers are referred to Bunch et al. (2016). PEG was used solely to examine quantifiable text features of responses—no automated scores were generated. While PEG’s custom feature set comprises nearly 1,000 linguistic variables, the features initially examined for the present study were limited to the approximately 80 interpretable and instructionally relevant variables used in PEG’s application in the Automated Writing Evaluation program *MI Write* (see Wilson et al., 2021). To identify a preliminary list of features, a supervised learning approach was used to identify those variables that best explained rater accuracy (i.e., variance in agreement between the rater scores and the expert scores assigned to validity responses). Specifically, a random forest classifier was used to rank the variables based on feature importance, as measured by mean decrease in impurity.^4^ With the goal of identifying a short list of variables that were reasonable from a human perspective, from this list I hand-selected eight variables to include in the model as fixed effects, considering three criteria: relative ranking, construct-relevance,^5^ and conceptual distinctiveness. Table 1 presents the eight text feature variables.

**Table 1:** Text feature variables.

Variable	Relative importance	Definition
Semantic precision	1	The percentage of words that are vague or general
Temporal connective word use	2	The percentage of words that suggest temporal connections between events
Lexical diversity	3	The percentage of total words in the response that are unique
Text cohesion	4	The number of times related words appear in consecutive sentences
Lexical sophistication	5	The percentage of words that show sophistication in word choice
Semantic accuracy	6	The percent of homophone errors in the response
Capitalization accuracy	7	The percentage of incorrectly capitalized proper nouns
Syntactic variety	8	The percentage of sentences that are not simple sentences

Relative importance is based on mean decrease in impurity.

Each item was aligned with a performance expectation that defined the specific knowledge, skill, or ability assessed by the item. The expectations effectively established the task, and thus influenced the nature of student responses. Three research expectations differentiated among analysis and integration of information, evaluation of information, and use of evidence. There were a total of nine writing expectations, each calling for writing or revising a short text’s (1) introduction, (2) conclusion, or (3) elaboration for one of three purposes: opinion/argumentative, narrative, or informational/explanatory. For purpose of analysis, I collapse the individual expectations into a dichotomous variable that distinguishes between research (0) and writing (1). Results include a sensitivity analysis that examines the potential loss of information from this approach. Developmental constraints on writing acquisition (Berninger and Swanson, 1994) implied that grade band would influence response content, thus I included fixed effects to allow for a comparison of reference grade band 6–8 with bands 3–5 and 11. Finally, the model included separate classifications and random effects for items and responses, described in further details in the *Analyses* section.

### Rater characteristics

Measures of rater characteristics included raters’ qualification performance, scoring experience, education, and current teaching experience.^6^ Two measures of qualification performance were included as fixed effects: the mean percent exact agreement with the benchmark scores of qualification sets and a dichotomous indicator of whether each rater produced any non-adjacent scores while qualifying on a given set. Raters were classified as new or inexperienced (i.e., experience on fewer than four scoring projects), experienced (four to eight projects), or senior (nine or more projects). Raters were classified by education level as holding undergraduate, graduate, or terminal degrees. I included a dichotomous variable to identify current teachers, who provided a current license. The model included a separate classification and random effects for raters.

### Scoring contexts

Scoring contexts examined included scoring location, shift, and team. For scoring location, a dichotomous variable identified whether raters worked remotely (0) or in a site-based location (1). Another dichotomous variable indicated whether each rater worked during a day (0) or a night shift (1). Scoring shifts were 6.5 h (day) and 3.75 h (night), excluding breaks. Day and evening shifts were available in EST, CST, PST, and HST zones; raters could select shift or zone regardless of their residence. Finally, the model included a separate classification and random effects for scoring teams.

### Analyses

**Figure 1:**
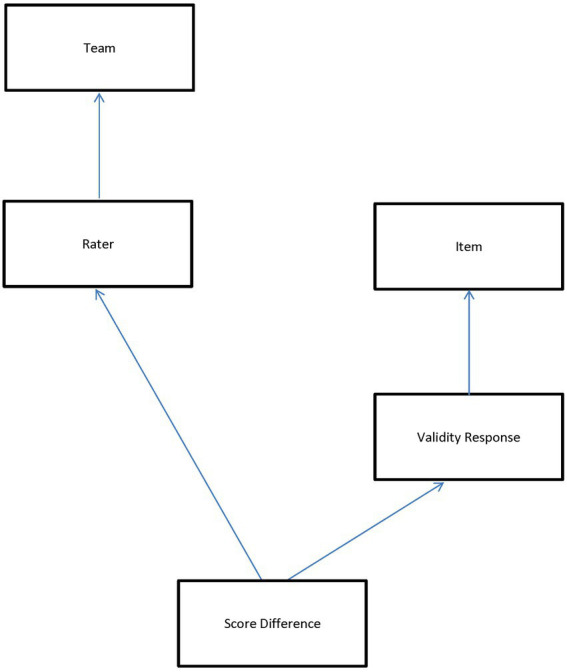
Cross-classification data diagram.

Rater monitoring data in large-scale assessment contexts seldom exhibit a perfectly hierarchical structure in which there is strict nesting of lower-level units within higher-level units. Instead, lower-level units are often nested within a cross-classification (or combination) of higher-level units. Figure 1 illustrates the non-hierarchical, multilevel structure of the present rater monitoring data using a classification diagram (Browne et al., 2001). Each classification unit is represented by a box; the classifications themselves are represented by arrows connecting the lowest level unit to the classification units. Boxes connected by an arrow indicate a nested relationship while unconnected boxes indicate a crossed relationship. The lowest level unit of Figure 1 is the absolute score difference between the rater-assigned score and the benchmark score associated with a validity response. Because multiple raters scored each validity response, score differences are shown nested within a cross-classification of raters and validity responses. Validity responses are nested in items, and each rater scored multiple items, so raters are also crossed with items. Finally, the top of the diagram shows raters nested in teams.

This data structure necessitated the specification of cross-classified multilevel models to accurately identify variance components and produce parameter estimates. Both frequentist and Bayesian methods have been applied to cross-classified models. However, Bayesian estimation has the advantage of greater computational efficiency for datasets with large numbers of units in each classification or those that deviate from a nested structure (Browne et al., 2001), both of which are true of the data analyzed in the present study. Bayesian estimation requires specification of a prior probability distribution (i.e., a prior) that reflects knowledge or uncertainty about each parameter. The prior distribution is updated using the data (i.e., the likelihood), resulting in a joint posterior distribution for the model parameters.

I fit cross-classified multilevel models using Bayesian Markov Chain Monte Carlo (MCMC) methods. MCMC takes iterative draws from the posterior distribution to explore and characterize the distribution of each parameter. Results are reported as means and standard deviations of the monitoring chains for each parameter, analogous to the parameter estimates and standard errors obtained in frequentist analyses.

Analyses involved fitting an increasingly complex series of models to address the study’s research questions. To first examine scoring accuracy variation across teams, items, raters, and responses, Model 1—an unconditional or null model—was used to simply decompose the total variation in accuracy into separate variance components. Expressed in classification notation (Browne et al., 2001), Model 1 took the form of:


(1) $$y_{i}=\beta_{0}+u_{team\left(i\right)}^{\left(5\right)}+u_{item\left(i\right)}^{\left(4\right)}+u_{rater\left(i\right)}^{\left(3\right)}+u_{validity\left(i\right)}^{\left(2\right)}+e_{i}$$

$$u_{team\left(i\right)}^{\left(5\right)}\sim N(0,\sigma_{u\left(5\right)}^{2})$$

$$u_{item\left(i\right)}^{\left(4\right)}\sim N(0,\sigma_{u\left(4\right)}^{2})$$

$$u_{rater\left(i\right)}^{\left(3\right)}\sim N(0,\sigma_{u\left(3\right)}^{2})$$

$$u_{validity\left(i\right)}^{\left(2\right)}\sim N(0,\sigma_{u\left(2\right)}^{2})$$

$$e_{i}\sim N(0,\sigma_{e}^{2})$$

Here, the dependent variable *y_i_* is *i*-th absolute score difference. The fixed part of the model contains only the intercept *β*_0_ which measures the overall mean (e.g., grand mean) of *y_i_* across all validity responses, raters, items, and teams. The random part of the model includes random effects classifications for teams, items, raters, and validity responses, as well as a residual error term for each absolute score difference. These random effects partition the variance in absolute score differences about *β*_0_ into sources of variation attributable to teams, items, raters, validity responses, and the residual, respectively. Random effects and residual errors are assumed independent and normally distributed with means of zero and constant variances; these assumptions were evaluated by examining (1) normal plots of standardized residuals and (2) standardized residuals plotted against predicted random effects.

Model 2 adds the time variable, the slope of which is allowed to vary randomly across raters. The time variable took the value of zero the first day a rater scored responses associated with a given item and increased by one each day. This predictor is used to measure the extent to which rater accuracy varied over time as well as the variability in raters’ linear time trends.


(2) $$\begin{array}{l} y_{i}=\beta_{0}+\beta_{1}{\mathrm{time}}_{i}+u_{team\left(i\right)}^{\left(5\right)}+u_{item\left(i\right)}^{\left(4\right)}+u_{0rater\left(i\right)}^{\left(3\right)}\\ \;\;\;+u_{1rater\left(i\right)}^{\left(3\right)}{\mathrm{time}}_{i}+u_{validity\left(i\right)}^{\left(2\right)}+e_{i} \end{array}$$

$$u_{team\left(i\right)}^{\left(5\right)}\sim N(0,\sigma_{u\left(5\right)}^{2})$$

$$u_{item\left(i\right)}^{\left(4\right)}\sim N(0,\sigma_{u\left(4\right)}^{2})$$

$$\left(\begin{array}{c} u_{0rater}^{\left(3\right)}\\ u_{1rater}^{\left(3\right)} \end{array}\right)\sim N\{(\begin{array}{c} 0\\ 0 \end{array}),\left(\begin{array}{cc} \sigma_{u0\left(3\right)}^{2} & \\ \sigma_{u01\left(3\right)}^{2} & \sigma_{u1\left(3\right)}^{2} \end{array}\right)\}$$

$$u_{validity\left(i\right)}^{\left(2\right)}\sim N(0,\sigma_{u\left(2\right)}^{2})$$

$$e_{i}\sim N(0,\sigma_{e}^{2})$$

Model 3 introduces response content fixed effects for the text features, assessment expectations, and grade bands.


(3) $$\begin{array}{l} y_{i}=\beta_{0}+\beta_{1}{\mathrm{time}}_{i}+\beta_{2}\mathrm{lexical}\_{\mathrm{diversity}}_{i}\\ \;\;+\beta_{3}\mathrm{syntactic}\_{\mathrm{variety}}_{i}\\ \;\;+\beta_{4}\mathrm{lexical}\_{\mathrm{sophistication}}_{i}\\ \;\;+\beta_{5}\mathrm{capitalization}\_{\mathrm{accuracy}}_{i}\\ \;\;+\beta_{6}\mathrm{semantic}\_{\mathrm{accuracy}}_{i}\\ \;\;+\beta_{7}\mathrm{temporal}\_{\mathrm{connective}}_{i}\\ \;\;+\beta_{8}\mathrm{semantic}\_{\mathrm{precision}}_{i}\\ \;\;+\beta_{9}\mathrm{text}\_{\mathrm{cohesion}}_{i}\\ \;\;+\beta_{10}\mathrm{writing}\_{\mathrm{expectation}}_{i}\\ \;\;+\beta_{11}\mathrm{grade}\_{\mathrm{band35}}_{i}\\ \;\;+\beta_{12}\mathrm{grade}\_{\mathrm{band11}}_{i}+u_{team\left(i\right)}^{\left(5\right)}+u_{item\left(i\right)}^{\left(4\right)}\\ \;\;+u_{0rater\left(i\right)}^{\left(3\right)}+u_{1rater\left(i\right)}^{\left(3\right)}{\mathrm{time}}_{i}+u_{validity\left(i\right)}^{\left(2\right)}+e_{i}\;\; \end{array}$$

$$u_{team\left(i\right)}^{\left(5\right)}\sim N(0,\sigma_{u\left(5\right)}^{2})$$

$$u_{item\left(i\right)}^{\left(4\right)}\sim N(0,\sigma_{u\left(4\right)}^{2})$$

$$\left(\begin{array}{c} u_{0rater}^{\left(3\right)}\\ u_{1rater}^{\left(3\right)} \end{array}\right)\sim N\{(\begin{array}{c} 0\\ 0 \end{array}),\left(\begin{array}{cc} \sigma_{u0\left(3\right)}^{2} & \\ \sigma_{u01\left(3\right)}^{2} & \sigma_{u1\left(3\right)}^{2} \end{array}\right)\}$$

$$u_{validity\left(i\right)}^{\left(2\right)}\sim N(0,\sigma_{u\left(2\right)}^{2})$$

$$e_{i}\sim N(0,\sigma_{e}^{2})$$

Based on Model 3 results, Model 4 is used to investigate whether the text feature variables interacted with the research/writing expectations.


(4) $$\begin{array}{l} y_{i}=\beta_{0}+\beta_{1}{\mathrm{time}}_{i}+\beta_{2}\mathrm{lexical}\_{\mathrm{diversity}}_{i}\\ \;\;\;+\beta_{3}\mathrm{syntactic}\_{\mathrm{variety}}_{i}+\beta_{4}\mathrm{lexical}\_{\mathrm{sophistication}}_{i}\\ \;\;\;\;\;\;\;\;\;\;\;\;\;+\beta_{5}capitalization\_accuracy_{i}\\ +\beta_{6}semantic\_accuracy_{i}\\ +\beta_{7}temporal\_connective_{i}\\ +\beta_{8}semantic\_precision_{i}+\beta_{9}text\_cohesion_{i}\\ +\beta_{10}writing\_expectation_{i}\\ +\beta_{11}grade\_band35_{i}+\beta_{12}grade\_band11_{i}\\ +\beta_{4}*\beta_{10}+\beta_{5}*\beta_{10}+\beta_{6}*\beta_{10}+\beta_{7}*\beta_{10}\\ \;\;\;\;\;\;\;\;\;\;\;\;+\beta_{8}*\beta_{10}+\beta_{9}*\beta_{10}+u_{team\left(i\right)}^{\left(5\right)}\\ \;\;\;\;\;\;\;\;\;\;\;\;+u_{item\left(i\right)}^{\left(4\right)}+u_{0rater\left(i\right)}^{\left(3\right)}+u_{1rater\left(i\right)}^{\left(3\right)}{\mathrm{time}}_{i}+u_{validity\left(i\right)}^{\left(2\right)}+e_{i} \end{array}$$

$$u_{team\left(i\right)}^{\left(5\right)}\sim N(0,\sigma_{u\left(5\right)}^{2})$$

$$u_{item\left(i\right)}^{\left(4\right)}\sim N(0,\sigma_{u\left(4\right)}^{2})$$

$$\left(\begin{array}{c} u_{0rater}^{\left(3\right)}\\ u_{1rater}^{\left(3\right)} \end{array}\right)\sim N\{(\begin{array}{c} 0\\ 0 \end{array}),\left(\begin{array}{cc} \sigma_{u0\left(3\right)}^{2} & \\ \sigma_{u01\left(3\right)}^{2} & \sigma_{u1\left(3\right)}^{2} \end{array}\right)\}$$

$$u_{validity\left(i\right)}^{\left(2\right)}\sim N(0,\sigma_{u\left(2\right)}^{2})$$

$$e_{i}\sim N(0,\sigma_{e}^{2})$$

Model 5 adds rater characteristic predictors for qualification performance, scoring experience, degree, and current teaching experience.


(5) $$\begin{array}{l} y_{i}=\beta_{0}+\beta_{1}time_{i}+\beta_{2}lexical\_diversity_{i}\\ +\beta_{3}syntactic\_variety_{i}\\ +\beta_{4}lexical\_sophistication_{i}\\ +\beta_{5}capitalization\_accuracy_{i}\\ +\beta_{6}semantic\_accuracy_{i}\\ +\beta_{7}temporal\_connective_{i}\\ +\beta_{8}semantic\_precision_{i}+\beta_{9}text\_cohesion_{i}\\ +\beta_{10}writing\_expectation_{i}\\ +\beta_{11}grade\_band35_{i}+\beta_{12}grade\_band11_{i}\\ +\beta_{7}*\beta_{10}+\beta_{8}*\beta_{10}+\beta_{9}*\beta_{10}\\ +\beta_{16}qualification\_exact_{i}\\ +\beta_{17}qualification\_nonadj_{i}\\ +\beta_{18}experienced_{i}+\beta_{19}senior_{i}\\ +\beta_{20}graduate\_degree_{i}+\beta_{21}terminal\_degree_{i}\\ +\beta_{22}current\_teacher_{i}+u_{team\left(i\right)}^{\left(5\right)}+u_{item\left(i\right)}^{\left(4\right)}+u_{0rater\left(i\right)}^{\left(3\right)}\\ \;\;\;\;\;\;\;\;\;\;\;\;+u_{1rater\left(i\right)}^{\left(3\right)}{\mathrm{time}}_{i}+u_{validity\left(i\right)}^{\left(2\right)}+e_{i} \end{array}$$

$$u_{team\left(i\right)}^{\left(5\right)}\sim N(0,\sigma_{u\left(5\right)}^{2})$$

$$u_{item\left(i\right)}^{\left(4\right)}\sim N(0,\sigma_{u\left(4\right)}^{2})$$

$$\left(\begin{array}{c} u_{0rater}^{\left(3\right)}\\ u_{1rater}^{\left(3\right)} \end{array}\right)\sim N\{(\begin{array}{c} 0\\ 0 \end{array}),\left(\begin{array}{cc} \sigma_{u0\left(3\right)}^{2} & \\ \sigma_{u01\left(3\right)}^{2} & \sigma_{u1\left(3\right)}^{2} \end{array}\right)\}$$

$$u_{validity\left(i\right)}^{\left(2\right)}\sim N(0,\sigma_{u\left(2\right)}^{2})$$

$$e_{i}\sim N(0,\sigma_{e}^{2})$$

Finally, Model 6 adds the scoring context variables for scoring location and scoring shift, which are treated as time-invariant covariates.


(6) $$\begin{array}{l} y_{i}=\beta_{0}+\beta_{1}{\mathrm{time}}_{i}+\beta_{2}\mathrm{lexical}\_{\mathrm{diversity}}_{i}\\ \;\;\;+\beta_{3}\mathrm{syntactic}\_{\mathrm{variety}}_{i}\\ \;\;\;+\beta_{4}\mathrm{lexical}\_{\mathrm{sophistication}}_{i}\\ \;\;\;+\beta_{5}\mathrm{capitalization}\_{\mathrm{accuracy}}_{i}\\ \;\;\;+\beta_{6}\mathrm{semantic}\_{\mathrm{accuracy}}_{i}\\ \;\;\;+\beta_{7}\mathrm{temporal}\_{\mathrm{connective}}_{i}\\ \;\;\;+\beta_{8}\mathrm{semantic}\_{\mathrm{precision}}_{i}\\ \;\;\;+\beta_{9}\mathrm{text}\_{\mathrm{cohesion}}_{i}\\ \;\;\;+\beta_{10}\mathrm{writing}\_{\mathrm{expectation}}_{i}\\ \;\;\;+\beta_{11}\mathrm{grade}\_\mathrm{band}35_{i}\\ \;\;\;+\beta_{12}\mathrm{grade}\_\mathrm{band}11_{i}+\beta_{7}*\beta_{10}\\ \;\;\;+\beta_{8}*\beta_{10}+\beta_{9}*\beta_{10}\\ \;\;\;+\beta_{16}\mathrm{qualification}\_{\mathrm{exact}}_{i}\\ \;\;\;+\beta_{17}\mathrm{qualification}\_{\mathrm{nonadj}}_{i}\\ \;\;\;+\beta_{18}{\mathrm{experienced}}_{i}+\beta_{19}{\mathrm{senior}}_{i}\\ \;\;\;+\beta_{20}\mathrm{graduate}\_{\mathrm{degree}}_{i}\\ \;\;\;+\beta_{21}\mathrm{terminal}\_{\mathrm{degree}}_{i}\\ \;\;\;+\beta_{22}\mathrm{current}\_{\mathrm{teacher}}_{i}\\ \;\;\;+\beta_{23}\mathrm{site}\_{\mathrm{based}}_{i}\\ \;\;\;+\beta_{24}\mathrm{evening}\_{\mathrm{shift}}_{i}+u_{team\left(i\right)}^{\left(5\right)}+u_{item\left(i\right)}^{\left(4\right)}\\ \;\;\;\;\;\;\;\;\;\;\;+u_{0rater\left(i\right)}^{\left(3\right)}+u_{1rater\left(i\right)}^{\left(3\right)}{\mathrm{time}}_{i}+u_{validity\left(i\right)}^{\left(2\right)}+e_{i} \end{array}$$

$$u_{team\left(i\right)}^{\left(5\right)}\sim N(0,\sigma_{u\left(5\right)}^{2})$$

$$u_{item\left(i\right)}^{\left(4\right)}\sim N(0,\sigma_{u\left(4\right)}^{2})$$

$$\left(\begin{array}{c} u_{0rater}^{\left(3\right)}\\ u_{1rater}^{\left(3\right)} \end{array}\right)\sim N\{(\begin{array}{c} 0\\ 0 \end{array}),\left(\begin{array}{cc} \sigma_{u0\left(3\right)}^{2} & \\ \sigma_{u01\left(3\right)}^{2} & \sigma_{u1\left(3\right)}^{2} \end{array}\right)\}$$

$$u_{validity\left(i\right)}^{\left(2\right)}\sim N(0,\sigma_{u\left(2\right)}^{2})$$

$$e_{i}\sim N(0,\sigma_{e}^{2})$$

All analyses were conducted using Bayesian estimation methods as implemented via MCMC procedures in MLwiN v3.05 (Rasbash et al., 2016; Browne, 2019). As I had no prior information about likely parameter values, I used the default prior distributions provided by MLwiN (see Browne, 2019). For all models, default priors were flat for all fixed parameters (i.e., p(*β*) ∝ 1) and minimally informative gamma for variance matrices (i.e., p(1/$\sigma_{e}^{2}$) ~ Gamma(0.001, 0.001)). Results include a sensitivity analysis that examines alternate prior specifications. Gibbs sampling was used to simulate a new value for each parameter in turn from its conditional or marginal distribution assuming that the current values for the other parameters are true values (Browne, 2019). All models were run for a burn-in of 50,000 iterations. The length of the monitoring portion of each chain was 500,000 iterations, storing every 10th iteration. Results are reported in the form of the posterior means, standard deviations, and 95% credible intervals of the parameter chains. Model fit is reported using the Deviance Information Criterion (DIC), which is a test statistic produced by the MCMC procedure based on model fit and complexity. Lower DIC values indicate better model fit (Spiegelhalter et al., 2002), and differences of five or more are considered substantial (Lunn et al., 2012).

Numerical and visual checks were conducted to confirm that the estimated results adequately described the posterior distribution and that the observed data were plausible under the models. For all models, MCMC diagnostics suggested that the sample was run for sufficiently long, based on a review of the autocorrelation function (ACF), the partial autocorrelation function (PACF), the Raftery–Lewis diagnostic,^7^ the Brooks–Draper diagnostic, and the effective sample size (ESS).

Appendix A provides further details of the checks of sample quality, examples, and results for each parameter of Model 6. Results of these checks showed, for each parameter, that the ESS exceeded 3,000, the Monte Carlo Standard Error was zero, and the Raftery-Lewis diagnostic was satisfied. Collectively, results provide strong evidence for convergence and a sufficient sample size.

## Results

### Scoring accuracy variation across teams, items, raters, responses, and time

Table 2 presents results of Models 1 and 2. Model 1 is the unconditional model which includes only an intercept, random effects (for teams, items, raters, and validity responses), and a residual error term. Ignoring time, this model estimates the overall mean absolute score difference to be 0.162. The random effects decompose the variation in absolute score differences into separate variance components attributable to teams, items, raters, validity responses, and residual error variation. To aid interpretation of the relative magnitude of the variance components, Table 3 shows the variance components translated to variance partition components, which report the proportion of the variance in absolute score differences associated with each classification unit.

**Table 2:** Parameter estimates for models 1 and 2.

	Model 1			Model 2		
	*M*	*SD*	95% CrI	*M*	*SD*	95% CrI
Fixed effects						
Intercept (*β*_0_)	0.162	0.005	0.152, 0.171	0.163	0.005	0.153, 0.172
Time (*β*_1_)				<−0.001	<0.001	−0.000, 0.000
Random effects						
Team variance ($\sigma_{u0\left(5\right)}^{2}$)	0.001	<0.001	0.000, 0.001	0.001	<0.001	0.000, 0.001
Item variance ($\sigma_{u0\left(4\right)}^{2}$)	0.004	<0.001	0.003, 0.005	0.004	<0.001	0.003, 0.005
Rater variance ($\sigma_{u0\left(3\right)}^{2}$)	0.004	<0.001	0.003, 0.004	0.004	<0.001	0.003, 0.004
Rater time slope covariance ($\sigma_{u01\left(3\right)}^{2}$)			<0.001, <0.001	<0.001	<0.001	<0.001, <0.001
Response variance ($\sigma_{u0\left(2\right)}^{2}$)	0.031	<0.001	0.030, 0.032	0.031	<0.001	0.030, 0.032
Residual variance ($\sigma_{e}^{2}$)	0.100	<0.001	0.100, 0.101	0.100	<0.001	0.100, 0.100
DIC	361999.8			359999.3		
DIC change				−2000.5		

M, posterior mean; SD, posterior standard deviation; CrI, credible interval of the posterior density estimate.

**Table 3:** Variance components and variance partition coefficients.

Classification	Variance component	Variance partition coefficient
Team	0.001	0.007
Item	0.004	0.029
Rater	0.004	0.029
Validity response	0.031	0.221
Residual	0.100	0.714
Total	0.140	1.000

**Figure 2:**
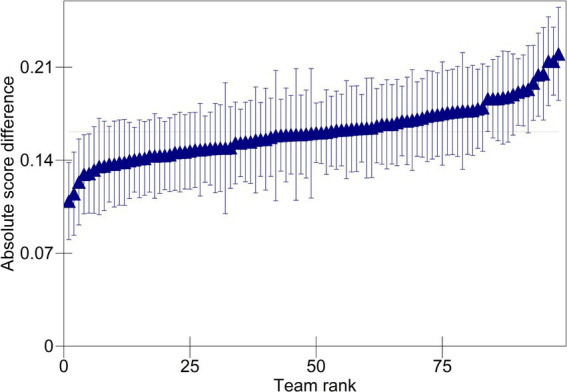
Predicted absolute score differences for teams.

The team variance (0.001) is small, accounting for only 0.71% of the total variation in absolute score differences. Figure 2 plots the estimated team effects. Triangles represent the mean absolute score difference for each team. Differences are centered around the median absolute score difference and plotted in ascending rank order. The corresponding 95% confidence intervals show whether the estimated team effects significantly differ from zero. The scale of the *y*-axis shows that, on average, teams had a small impact on accuracy. The confidence intervals in the plot cross the zero line in most cases, indicating the estimated effects of most teams do not differ significantly from zero. Specifically, of the 98 teams, five were more accurate than average (seen on the left of the plot), and nine were less accurate than average (seen on the right of the plot).

**Figure 3:**
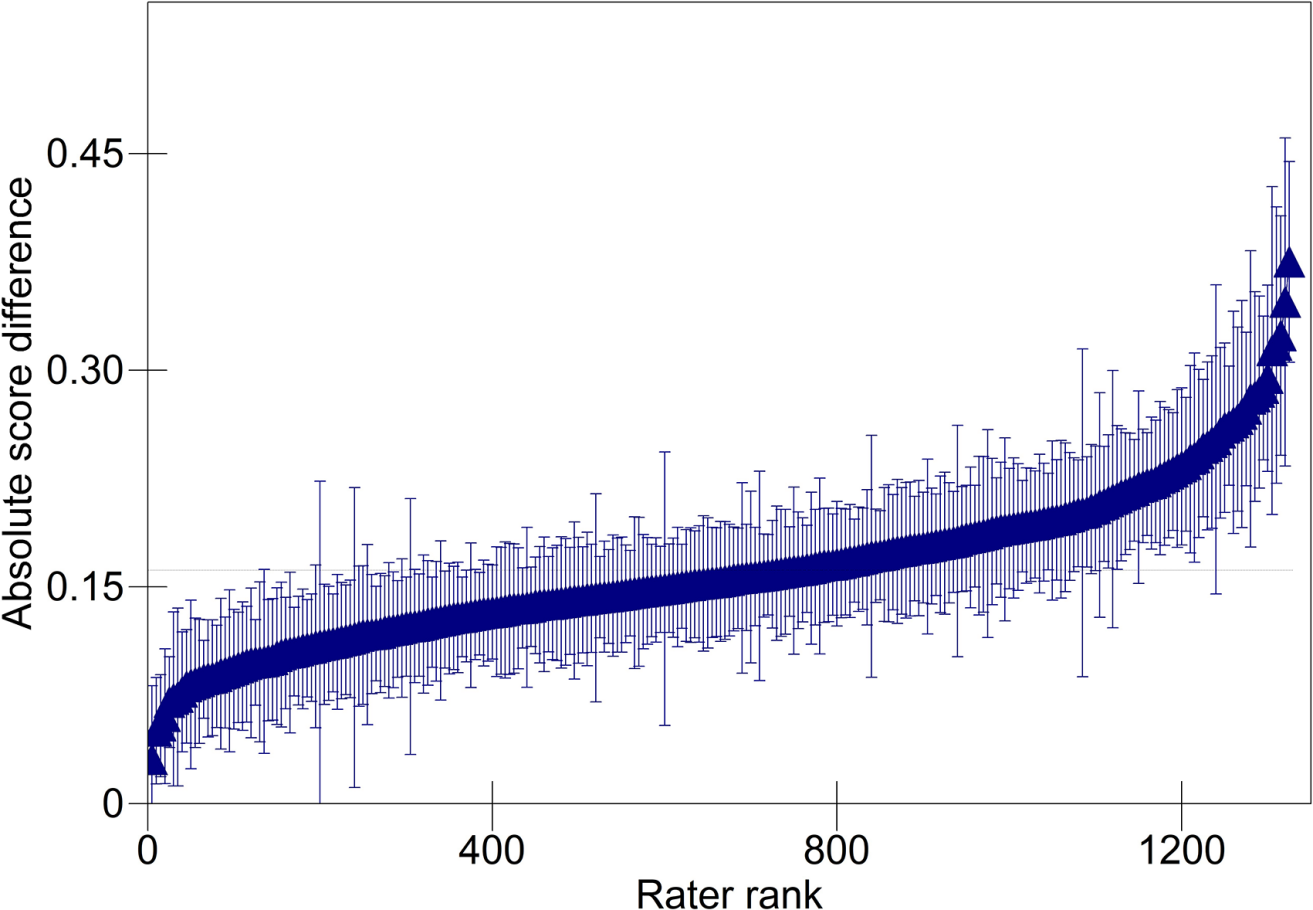
Predicted absolute score differences for raters. For clarity, every 5th rater is plotted.

The item and rater variances are, respectively, larger (both 0.004); however, each one accounts for only 2.86% of the total variation in absolute score differences. Figure 3 plots the estimated rater effects. While the range of the *y*-axis is increased compared to that of Figure 2, the range seen in the scale of the *y*-axis shows relatively little variation in accuracy across raters within teams and indicates that raters, on average, had a small impact on accuracy. The estimated effects of most raters do not differ significantly from zero. Of the 1,329 raters, 273 were more accurate than the average rater, and 221 were less accurate than average.

The response variance (0.031) is much larger, and accounts for 22.14% of the total variation in absolute score differences. Adjusting for team, item, and rater effects, there remained considerable differences in accuracy attributable to individual validity responses. Finally, the residual variance (0.100) accounts for 71.43% of the total variation in absolute score differences. Consistent with previous examinations of scoring accuracy (e.g., Leckie and Baird, 2011; Baird et al., 2017), this is the largest source of variation, reflecting unexamined characteristics of raters, responses, and the interaction between them.

**Figure 4:**
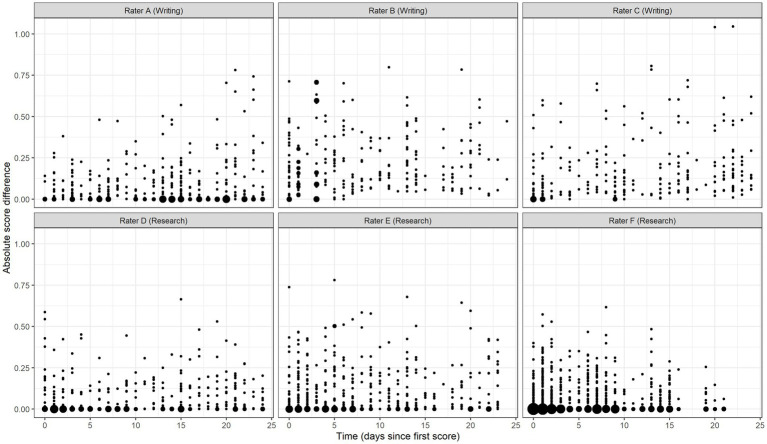
Predicted absolute score differences over time for six random raters.

Model 2 adds the time variable to assess the change in accuracy over time. Results show an estimated intercept (*β*_0_) value of 0.163 (95% CrI = [0.153, 0.172]), reflecting the mean absolute score difference across raters on the day that each rater scored their first validity response. This value decreased nominally each subsequent day of scoring (*β*_1_ = <−0.001, 95% CrI = [−0.000, 0.000]), thus there is no evidence of substantive change in the degree of raters’ collective scoring accuracy over time. However, in the random part of the model, the rater time slope covariance estimate indicates intra-rater variation in accuracy over time ($\sigma_{u01\left(3\right)}^{2}$ = <0.001, 95% CrI = [<0.001, <0.001]). To facilitate interpretation, Figure 4 plots the predicted absolute score differences for six random raters across 25 days of scoring. In this figure, the size of each point is proportional to the number of observations at the location. This figure shows that individual raters demonstrated considerable variation in scoring accuracy from response to response and from day to day. Compared to Model 1, Model 2 provides significantly better fit to the data (ΔDIC = −2000.5).

### Influence of response content, rater characteristics, and scoring context on scoring accuracy

#### Response content

Table 4 presents results of Models 3 and 4. Model 3 adds the response content variables associated with text features, assessment expectations, and grade bands. Of the eight text feature variables, four (syntactic variety, lexical sophistication, temporal connective word use, and text cohesion) are positively associated with absolute score differences, indicating that responses exhibiting a greater proportion of these features were more difficult for raters to score. The remaining four text feature variables (lexical diversity, capitalization accuracy, semantic accuracy, and semantic precision) are negatively related to the dependent variable, meaning that responses with a greater prevalence of these features were easier to score.

**Table 4:** Parameter estimates for models 3 and 4.

Parameter	Model 3			Model 4		
	*M*	*SD*	95% CrI	*M*	*SD*	95% CrI
Fixed effects						
Intercept (*β*_0_)	0.135	0.007	0.121, 0.149	0.134	0.007	0.120, 0.148
Time (*β*_1_)	<−0.001	<0.001	−0.000, 0.000	<−0.001	<0.001	−0.000, 0.000
*Response Content*						
Lexical diversity (*β*_2_)	−0.265	0.013	−0.291, −0.239	−0.264	0.014	−0.292, −0.237
Syntactic variety (*β*_3_)	0.669	0.124	0.426, 0.912	0.624	0.130	0.367, 0.875
Lexical soph. (*β*_4_)	0.769	0.186	0.402, 1.133	0.682	0.190	0.307, 1.052
Cap. accuracy (*β*_5_)	−0.453	0.120	−0.691, −0.218	−0.510	0.133	−0.767, −0.250
Semantic accuracy (*β*_6_)	−0.650	0.082	−0.810, −0.489	−0.658	0.087	−0.827, −0.488
Temporal conn. use (*β*_7_)	0.219	0.079	0.064, 0.373	0.082	0.089	−0.091, 0.257
Semantic precision (*β*_8_)	−0.154	0.026	−0.205, −0.104	−0.207	0.027	−0.261, −0.153
Text cohesion (*β*_9_)	0.022	0.003	0.017, 0.028	0.027	0.003	0.022,0.033
Research expectation	Ref			Ref		
Writing expectation (*β*_10_)	0.044	0.009	0.026, 0.062	0.043	0.009	0.025, 0.061
Grade band 3–5 (*β*_11_)	0.025	0.008	0.009, 0.041	0.024	0.008	0.008, 0.040
Grade band 6–8	Ref			Ref		
Grade band 11 (*β*_12_)	−0.001	0.010	−0.020, 0.018	−0.000	0.010	−0.019, 0.019
Lexical diversity*Writing exp. (*β*_13_)				−0.010	0.017	−0.043, 0.024
Syntactic variety*Writing exp. (*β*_14_)				0.087	0.156	−0.219, 0.396
Lexical soph.*Writing exp. (*β*_15_)				0.454	0.276	−0.082, 1.001
Cap. accuracy*Writing exp. (*β*_16_)				0.188	0.148	−0.102, 0.476
Semantic accuracy*Writing exp. (*β*_17_)				0.049	0.094	−0.137, 0.234
Temporal conn. use*Writing exp. (*β*_18_)				0.340	0.101	0.142, 0.539
Semantic precision*Writing exp. (*β*_19_)				0.196	0.032	0.133, 0.259
Text cohesion*Writing exp. (*β*_20_)				−0.026	0.004	−0.033, −0.018
Random effects						
Team variance ($\sigma_{u0\left(5\right)}^{2}$)	0.001	<0.001	0.000, 0.001	0.001	<0.001	0.000, 0.001
Item variance ($\sigma_{u0\left(4\right)}^{2}$)	0.004	<0.001	0.003, 0.005	0.004	<0.001	0.003, 0.005
Rater variance ($\sigma_{u0\left(3\right)}^{2}$)	0.004	<0.001	0.003, 0.004	0.004	<0.001	0.003, 0.004
Rater time slope covariance ($\sigma_{u01\left(3\right)}^{2}$)	<0.001	<0.001	<0.001, <0.001	<0.001	<0.001	<0.001, <0.001
Response variance ($\sigma_{u0\left(2\right)}^{2}$)	0.027	<0.001	0.027, 0.028	0.027	<0.001	0.027, 0.028
Residual variance ($\sigma_{e}^{2}$)	0.100	<0.001	0.100, 0.100	0.100	<0.001	0.100, 0.100
DIC	359887.1			359770.8		
DIC change	−112.2			−116.3		

M, posterior mean; SD, posterior standard deviation; CrI, credible interval of the posterior density estimate.

Response content results further showed that writing content was more difficult to score than research content (*β*_10_ = 0.044, 95% CrI = [−0.026, 0.062]). Raters found content produced by grade 3–5 students slightly more difficult to score than content produced by grade 6–8 or high school students (*β*_11_ = 0.025, 95% CrI = [0.009, 0.041]). The addition of the response content variables explains 13% of the response-level variance ($\sigma_{u0\left(2\right)}^{2}$) observed in Model 2 [(0.031–0.027)/0.031] and improves fit to the data (ΔDIC = −112.2).

**Figure 5:**
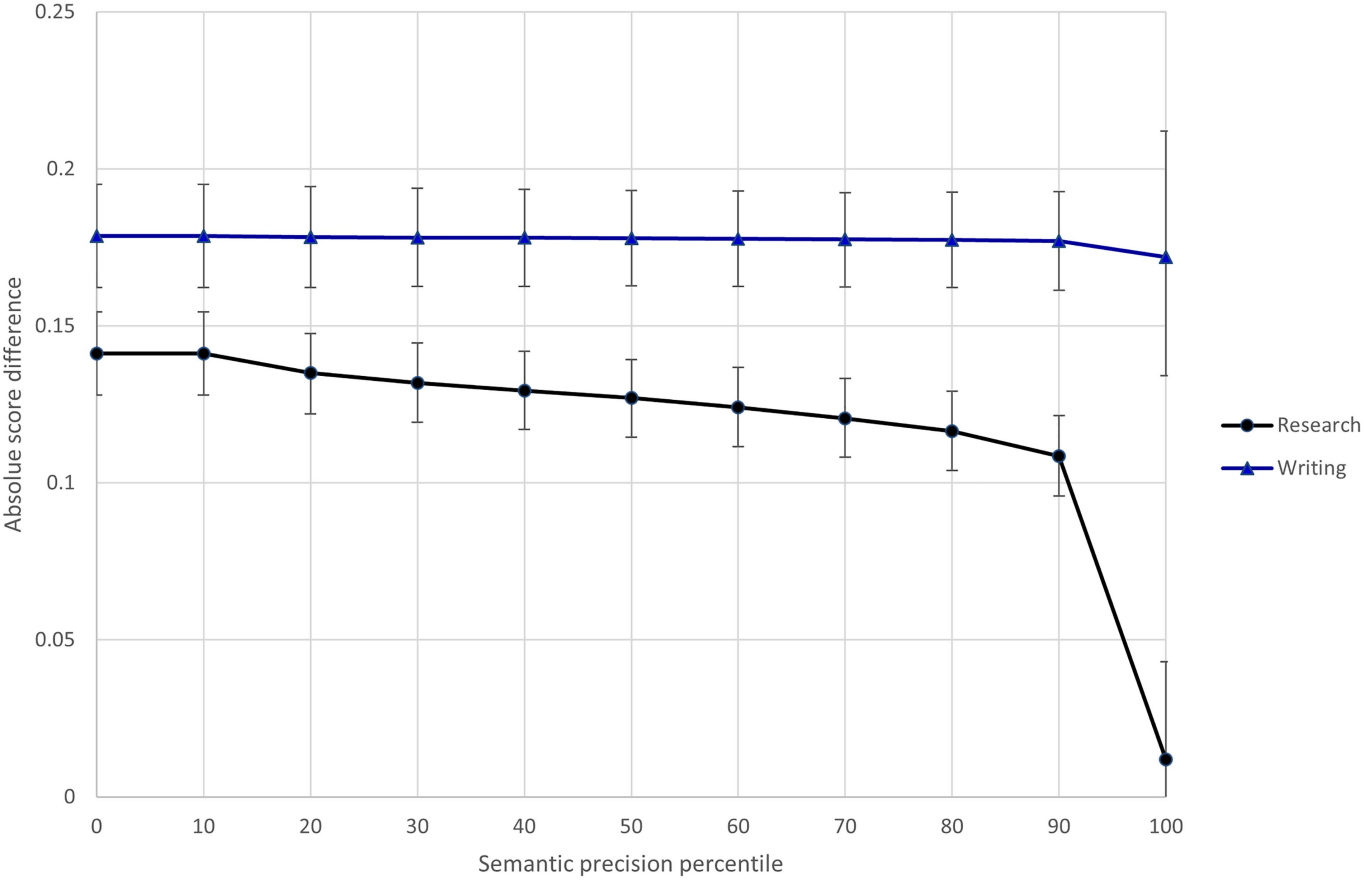
Differential influence of response semantic precision on difficulty of scoring research and writing content.

**Figure 6:**
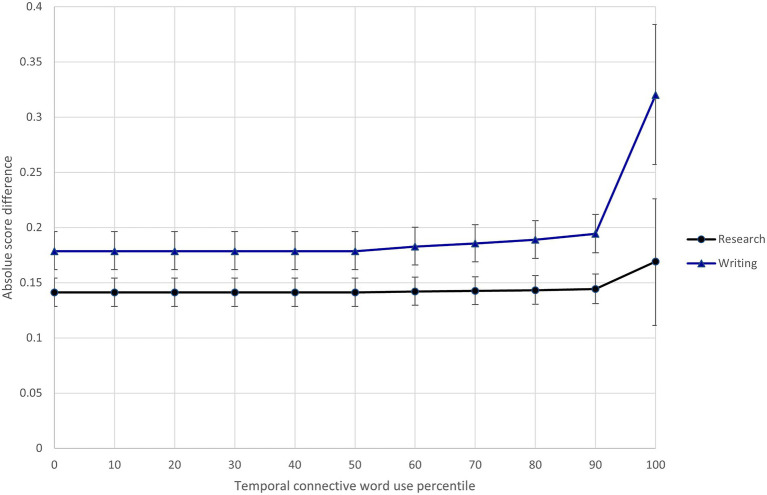
Differential influence of response temporal connective word use on difficulty of scoring research and writing content.

**Figure 7:**
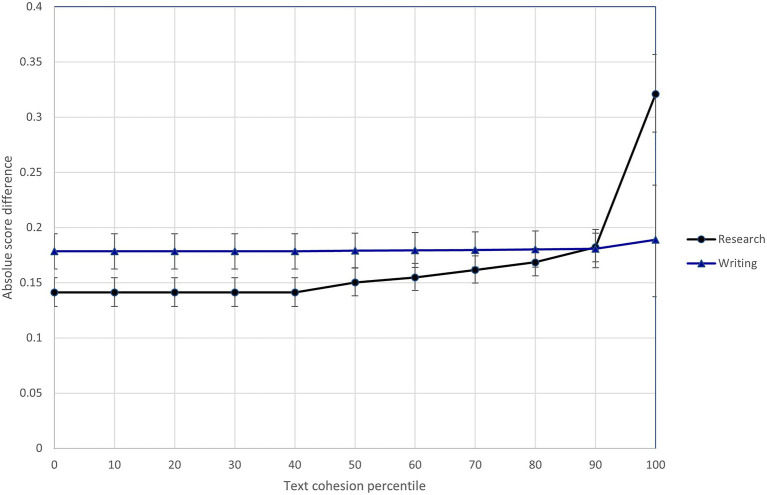
Differential influence of response text cohesion on difficulty of scoring research and writing content.

In light of Model 3 results, I specified Model 4 to investigate whether the text feature variables interacted with the research/writing expectations. Results of this model show that three of the text features (semantic precision, temporal connective word use, and text cohesion) differentially influenced the difficulty of scoring research and writing content.^8^ To illustrate these interactions, Figures 5–7 show the relationship between predicted score differences and presence of text features for research and writing content separately. Adding the interactions to the model improved model fit (ΔDIC = −116.3).

#### Rater characteristics

Table 5 presents results of Models 5 and 6. Model 5 adds the rater characteristics variables for qualification performance, scoring experience, degree, and current teaching experience. Recall that two predictors were used to examine qualification performance: agreement with the benchmark scores and assignment of a non-adjacent score. Results showed that raters who were more accurate during qualification were slightly more accurate during operational scoring; each percentage increase in mean exact agreement (*β*_16_) was associated with a decrease in absolute score difference of <−0.001 (95% CrI = [−0.001, −0.000]). Raters who produced non-adjacent scores during qualification (*β*_17_) had absolute score differences that were on average 0.02 points greater than raters who did not produce non-adjacent scores during qualification (95% CrI = [0.013, 0.028]). Despite passing a second qualification set and thus ultimately meeting the qualification criteria, raters who produced non-adjacent scores during qualification were less accurate, on average, than peers with comparable qualification exact agreement.

**Table 5:** Parameter estimates for models 5 and 6.

Parameter	Model 5			Model 6		
	*M*	*SD*	95% CrI	*M*	*SD*	95% CrI
Fixed effects						
Intercept (*β*_0_)	0.133	0.007	0.118, 0.147	0.125	0.008	0.110, 0.141
Time (*β*_1_)	<−0.001	<0.001	−0.000, 0.000	<−0.001	<0.001	−0.000, 0.000
*Response Content*						
Lexical diversity (*β*_2_)	−0.267	0.013	−0.293, −0.241	−0.267	0.013	−0.293, −0.241
Syntactic variety (*β*_3_)	0.639	0.126	0.392, 0.885	0.641	0.125	0.394, 0.885
Lexical soph. (*β*_4_)	0.758	0.186	0.393, 1.122	0.758	0.187	0.391, 1.123
Cap. accuracy (*β*_5_)	−0.445	0.120	−0.680, −0.211	−0.444	0.119	−0.677, −0.209
Semantic accuracy (*β*_6_)	−0.647	0.082	−0.806, −0.486	−0.648	0.082	−0.807, −0.488
Temporal conn. use (*β*_7_)	0.081	0.089	−0.094, 0.255	0.081	0.089	−0.094, 0.255
Semantic precision (*β*_8_)	−0.206	0.027	−0.259, −0.152	−0.206	0.027	−0.259, −0.152
Text cohesion (*β*_9_)	0.027	0.003	0.022, 0.032	0.027	0.003	0.021, 0.032
Research expectation	Ref			Ref		
Writing expectation (*β*_10_)	0.043	0.009	0.025, 0.061	0.044	0.011	0.026, 0.063
Grade band 3–5 (*β*_11_)	0.025	0.008	0.009, 0.041	0.025	0.008	0.008, 0.041
Grade band 6–8	Ref			Ref		
Grade band 11 (*β*_12_)	−0.004	0.010	−0.023, 0.015	−0.004	0.010	−0.023, 0.015
Temporal conn. use*Writing exp. (*β*_13_)	0.339	0.100	0.144, 0.534	0.340	0.100	0.142, 0.537
Semantic precision*Writing exp. (*β*_14_)	0.191	0.032	0.128, 0.252	0.190	0.032	0.129, 0.252
Text cohesion*Writing exp. (*β*_15_)	−0.024	0.003	−0.031, −0.018	−0.024	0.003	−0.031, −0.018
*Rater Characteristics*						
Qual percent exact (*β*_16_)	<−0.001	<0.001	−0.001, −0.000	<−0.001	<0.001	−0.001, −0.000
Qual non-adjacent (*β*_17_)	0.021	0.004	0.013, 0.028	0.020	0.004	0.013, 0.028
New/inexperienced	Ref			Ref		
Experienced (*β*_18_)	−0.004	0.007	−0.019, 0.010	−0.007	0.007	−0.022, 0.007
Senior (*β*_19_)	−0.008	0.008	−0.024, 0.007	−0.011	0.008	−0.027, 0.005
Undergraduate degree	Ref			Ref		
Graduate degree (*β*_20_)	0.004	0.004	−0.003, 0.012	0.004	0.004	−0.003, 0.012
Terminal degree (*β*_21_)	0.014	0.010	−0.006, 0.034	0.014	0.010	−0.006, 0.034
Current teacher (*β*_22_)	−0.011	0.008	−0.027, 0.005	−0.013	0.008	−0.029, 0.003
*Scoring Context*						
Site based (*β*_23_)				0.008	0.005	−0.002, 0.019
Evening shift (*β*_24_)				0.012	0.006	0.001, 0.023
Random effects						
Team variance ($\sigma_{u0\left(5\right)}^{2}$)	0.001	<0.001	0.000, 0.001	0.001	<0.001	0.000, 0.001
Item variance ($\sigma_{u0\left(4\right)}^{2}$)	0.004	<0.001	0.003, 0.005	0.004	<0.001	0.003, 0.005
Rater variance ($\sigma_{u0\left(3\right)}^{2}$)	0.004	<0.001	0.003, 0.004	0.004	<0.001	0.003, 0.004
Rater time slope covariance ($\sigma_{u01\left(3\right)}^{2}$)	<0.001	<0.001	<0.001, <0.001	<0.001	<0.001	<0.001, <0.001
Response variance ($\sigma_{u0\left(2\right)}^{2}$)	0.027	<0.001	0.027, 0.028	0.027	<0.001	0.027, 0.028
Residual variance ($\sigma_{e}^{2}$)	0.100	<0.001	0.100, 0.100	0.100	<0.001	0.100, 0.100
DIC	359748.9			359751.9		
DIC change	−21.9			3.0		

M, posterior mean; SD, posterior standard deviation; CrI, credible interval of the posterior density estimate.

Neither rater experience nor rater education level had an effect on absolute score differences. Being a current teacher did not affect absolute score differences, suggesting that teachers and non-teachers scored with a similar level of accuracy. Collectively, the addition of the rater characteristics improved model fit compared to Model 4 (ΔDIC = −21.9).

#### Scoring contexts

Model 6 adds scoring context covariates for scoring location and scoring shift. Results showed no estimated differences in scoring accuracy between remote and site-based raters (*β*_23_ = 0.008, 95% CrI = [−0.002, 0.019]). There was a small influence of scoring shift on accuracy (*β*_24_); raters who worked evening shifts had absolute score differences that were on average 0.012 points greater than raters who worked during the day (95% CrI = [0.001, 0.023]). Overall, however, the addition of the scoring context variables did not lead to an improvement in model fit (ΔDIC = 3.0).

#### Sensitivity analyses

The choice of default priors introduces some risk that the default priors do not accurately represent the prior information. I thus re-estimated Model 6 using several different priors to examine the sensitivity of the results to the default prior distributions used in estimating the model. I examined two alternate prior distributions, specifically, (1) alternate minimally informative gamma for variance matrices (i.e., p(1/$\sigma_{e}^{2}$) ~ Gamma(1.0, 0.001)) and (2) uniform on variance scale priors. Table 6 presents results of this sensitivity analysis. The alternate prior specifications show, overall, that the posterior distribution was robust to the choice of prior distribution. Specifically, parameter estimates (means and standard deviations) all agreed to at least two decimal places under all three prior specifications. These results indicate that the priors did not have undue influence on estimates.

**Table 6:** Effects of alternate prior specification on parameter estimates for model 6.

Parameter	Default priors		Alternative gamma priors		Uniform on variance scale priors	
	*M*	*SD*	*M*	*SD*	*M*	*SD*
Fixed effects						
Intercept (*β*_0_)	0.125	0.008	0.125	0.008	0.125	0.008
Time (*β*_1_)	<−0.001	<0.001	<−0.001	<0.001	<−0.001	<0.001
*Response Content*						
Lexical diversity (*β*_2_)	−0.267	0.013	−0.267	0.013	−0.267	0.013
Syntactic variety (*β*_3_)	0.641	0.125	0.641	0.125	0.639	0.125
Lexical soph. (*β*_4_)	0.758	0.187	0.757	0.185	0.759	0.185
Cap. accuracy (*β*_5_)	−0.444	0.119	−0.442	0.120	−0.445	0.119
Semantic accuracy (*β*_6_)	−0.648	0.082	−0.647	0.082	−0.647	0.082
Temporal con. use (*β*_7_)	0.081	0.089	0.080	0.089	0.082	0.088
Semantic precision (*β*_8_)	−0.206	0.027	−0.205	0.027	−0.205	0.027
Text cohesion (*β*_9_)	0.027	0.003	0.027	0.003	0.027	0.003
Research expectation	Ref		Ref		Ref	
Writing expectation (*β*_10_)	0.044	0.011	0.044	0.010	0.045	0.010
Grade band 3–5 (*β*_11_)	0.025	0.008	0.024	0.008	0.024	0.008
Grade band 6–8	Ref		Ref		Ref	
Grade band 11 (*β*_12_)	−0.004	0.010	−0.004	0.01	−0.004	0.010
Temporal con. use*Writing exp. (*β*_13_)	0.340	0.100	0.341	0.10	0.340	0.100
Semantic precision*Writing exp. (*β*_14_)	0.190	0.032	0.190	0.032	0.190	0.031
Text cohesion*Writing exp. (*β*_15_)	−0.024	0.003	−0.024	0.003	−0.024	0.003
*Rater Characteristics*						
Qual percent exact (*β*_16_)	<−0.001	<0.001	<−0.001	<0.001	<−0.001	<0.001
Qual non-adjacent (*β*_17_)	0.020	0.004	0.020	0.004	0.020	0.004
New/inexperienced	Ref		Ref		Ref	
Experienced (*β*_18_)	−0.007	0.007	−0.007	0.007	−0.007	0.007
Senior (*β*_19_)	−0.011	0.008	−0.011	0.008	−0.011	0.008
Undergraduate degree	Ref		Ref		Ref	
Graduate degree (*β*_20_)	0.004	0.004	0.004	0.004	0.004	0.004
Terminal degree (*β*_21_)	0.014	0.010	0.014	0.010	0.014	0.010
Current teacher (*β*_22_)	−0.013	0.008	−0.013	0.008	−0.013	0.008
*Scoring Context*						
Site based (*β*_23_)	0.008	0.005	0.008	0.005	0.009	0.005
Evening shift (*β*_24_)	0.012	0.006	0.012	0.006	0.012	0.006
Random effects						
Team variance ($\sigma_{u0\left(5\right)}^{2}$)	0.001	<0.001	0.001	<0.001	0.001	<0.001
Item variance ($\sigma_{u0\left(4\right)}^{2}$)	0.004	<0.001	0.004	<0.001	0.004	<0.001
Rater variance ($\sigma_{u0\left(3\right)}^{2}$)	0.004	<0.001	0.004	<0.001	0.004	<0.001
Rater time slope covariance ($\sigma_{u01\left(3\right)}^{2}$)	<0.001	<0.001	<0.001	<0.001	<0.001	<0.001
Response variance ($\sigma_{u0\left(2\right)}^{2}$)	0.027	<0.001	0.027	<0.001	0.027	<0.001
Residual variance ($\sigma_{e}^{2}$)	0.100	<0.001	0.100	<0.001	0.100	<0.001

M, posterior mean; SD, posterior standard deviation.

As an additional sensitivity analysis, I examined the potential loss of information due to combining the three research and nine writing expectations into a single category for each as part (see Appendix B). Using a single research expectation as the reference group, I find significant variability in absolute score differences across expectations, but a similar pattern of results to the more parsimonious model. Specifically, compared to the reference research expectation (requiring analysis and integration of information), response content associated with the other two research expectations (requiring evaluation of information and use of evidence) were slightly easier to score. The majority (seven of the nine) writing expectations were more difficult to score than the reference research expectation. Finally, raters found content in which students wrote/revised an introduction or conclusion to an informative/explanatory text most difficult to score. Full results of this additional sensitivity analysis are presented in Appendix B.

## Discussion

Cross-classified multilevel model results showed that relatively little variation in scoring accuracy was attributable to teams (1%), items (3%), and raters (3%), while validity responses accounted for 22% of the total variation in accuracy. On average, raters did not collectively exhibit differential accuracy over time, though there was significant variation in individual rater’s scoring accuracy from response to response and day to day. The considerable response-level variation in accuracy was explained in part by the content of students’ responses; I identified specific text features, research/writing content, and student grade bands that contributed to scoring difficulty. Also, some text features differentially influenced the difficulty of scoring research and writing content. Multiple measures of raters’ qualification performance predicted their scoring accuracy, but general rater background characteristics including experience and education did not. Finally, site-based and remote raters demonstrated comparable accuracy, while evening-shift raters were slightly less accurate, on average, than day-shift raters. As the first naturalistic, integrated examination of rater accuracy in the context of a large-scale assessment program, the present study extends previous research and provides implications for rater recruitment, training, monitoring, and feedback to improve human evaluation of written responses.

### Scoring accuracy variation across teams, items, raters, responses, and time

Results showed notable team, item, and rater effects on scoring accuracy. Collectively, however, these effects comprised only 6.4% of the total variation in absolute score differences. The relatively small magnitude of these effects likely suggests the rater training, qualification, and monitoring processes employed in the assessment program adequately mitigated systematic differences in accuracy that might have otherwise been attributed to teams, items, and raters. Consistent with previous research, individual responses had a considerable influence on scoring accuracy (Leckie and Baird, 2011; Baird et al., 2013; Zhao et al., 2017).

I found no substantive evidence of systematic change in average rater accuracy over time, though individual raters’ predicted score differences varied significantly about the average linear time trend. These findings accord with prior studies that have shown intra-rater differences in accuracy over time (Myford and Wolfe, 2009; Leckie and Baird, 2011; Baird et al., 2013). This within-rater variability has been hypothesized to be due to (unexamined) interaction effects between raters and responses (Leckie and Baird, 2011). While results of the present study provide new evidence of the contribution of individual responses to scoring difficulty, additional, unexamined factors contributing to within-rater inconsistency likely remain. Future research should continue efforts to explicate factors contributing to within-rater variability to better improve rater training and monitoring systems.

The content of responses had a considerable impact on scoring accuracy, adjusting for rater, item, and team effects. I identified text features of responses that facilitated scoring accuracy, including unique words (semantic accuracy) and capitalization errors (capitalization accuracy), as well as text features that hindered scoring accuracy, including sophisticated word choice (lexical sophistication) and varied sentences (syntactic variety). These results extend prior research by situating the influence of text features on scoring accuracy within the context of broader antecedents of rater effects.

Accounting for text features, both indirect measures of response content—assessment expectations and grade bands to which items were aligned—proved to influence scoring accuracy. There are two likely explanations for the finding that writing content was more difficult to score, on average, than research content. Recall that raters evaluated research responses using item-specific rubrics; these rubrics defined the requisite content needed for a full-credit response as well as the quality and quantity of evidence or support required for partial credit within the context of each item, making their interpretation relatively straightforward. Raters evaluated writing responses, on the other hand, using rubrics that were generic for each writing purpose and grade band. Because the generic writing rubrics were, by necessity, less prescriptive than the item-specific research rubrics, the writing rubrics provided raters with less direct guidance for evaluating response content, which likely resulted in the writing responses being more difficult to score. A second possible, related explanation is that evaluating the writing quality of a response, which represents a continuum, was an inherently more subjective exercise than evaluating analysis of information or use of evidence. Put another way, there was particular content that made a research response objectively right or wrong. The same was not true of writing responses, which may explain why raters were able to score research responses accurately with relatively greater ease.

Three text features (semantic precision, temporal connective word use, and text cohesion) interacted with the assessment expectations to differentially influence the difficulty of scoring research and writing content. Raters found the more semantically imprecise research responses (shown in the 80th + percentiles in Figure 5) much easier to score than research responses exhibiting average semantic precision. At the same time, semantic precision had little impact on the ease of scoring writing content. The opposite pattern was seen for temporal connective word use, where a relative high degree of temporal connective word use (shown in the 90th + percentiles in Figure 6) made writing content much more difficult to score, but much less so research content. Finally, the difficulty of scoring writing content was largely insensitive to the text cohesion measure, while there was a point at which (shown by the 90th + percentiles in Figure 7), research content that included a high degree of related words in consecutive sentences became more difficult to score than similarly cohesive writing content. Overall, these results provide further evidence of the impact of text features on scoring difficultly and the moderating effect of content-specific scoring criteria.

Adjusting for item effects, I found responses composed by grade 3–5 students more challenging to score than those composed by grade 6–8 or 11 students. This is likely due to the influence of younger students’ developing translation (i.e., text generation and transcription) skills on the content of their responses (Berninger and Swanson, 1994). Compared to more mature writers, younger students tended to generate more idiosyncratic responses which were less likely to clearly align with the rubrics and anchor responses. Additionally, grade 3–5 students were more likely than older students to have constraints in transcription ability that impeded raters’ comprehension of their responses.

### Rater characteristics

Raters’ performance characteristics proved to be better predictors of accuracy than raters’ background characteristics. There was modest evidence that rater certification worked as intended, as qualification performance was associated with operational scoring accuracy. Furthermore, raters who produced a non-adjacent score during qualification, all other things being equal, were less accurate during operational scoring. Thus, it appears that a non-adjacent score during qualification may function as an indicator that a rater has not grasped the scoring criteria quite as well as their peers, regardless of overall qualification performance.

Rater background characteristics, including scoring experience, education level, and current teaching experience, had no impact on scoring accuracy. These results, controlling for a host of other determinates of accuracy and estimated across a large pool of raters, extend prior research (e.g., Leckie and Baird, 2011; Song et al., 2014) suggesting that rater background has little influence on rating quality.

### Scoring contexts

Scoring contexts had mixed effects on scoring accuracy. Site-based and remote raters exhibited comparable accuracy, as suggested by previous research (e.g., Wolfe et al., 2010; Knoch et al., 2018). However, raters who worked during evening shifts were found to be slightly less accurate than those who worked during the day, all else equal. This is likely due to the shorter length of night shifts, which provide raters less uninterrupted time and practice than day shifts. Night shift raters may have also suffered greater fatigue, particularly those for whom scoring was a second job.

### Limitations

Study results were based on analysis of a secondary dataset and are thus subject to several limitations. I analyzed rater monitoring data exclusively and not scores assigned to operational student responses. It is likely that the monitoring data included some scores assigned by raters who were ultimately terminated based on performance (and thus would not contribute to operational results during some or all of their tenure). Qualification performance results were limited by the sample, which excluded raters who failed to qualify or otherwise did not score operationally. I examined only current teaching experience; thus, results may not apply to all former teachers. This study examined validity data associated with a single administration of one assessment program and results may not generalize across administrations or to other programs.

### Implications and future directions

Results of the present study offer several implications for practice. Knowledge of the relative impact of the factors that influence rater accuracy can be used to establish evidence-based policy in state assessment programs. Some current state policies, such as those requiring experienced raters, teaching experience, or site-based scoring, increase the cost and complexity of an assessment program^9^ and may not produce the anticipated improvements in score quality. Such policies should be reviewed in light of this new evidence to ensure there is an empirical justification for them, particularly while labor shortages due to the post-COVID-19 pandemic recovery exacerbate the challenges associated with recruiting large numbers of raters.

Moreover, results can inform more strategic investments by scoring service providers to improve score quality. For example, findings suggest a greater return on investment could be expected from allocating resources to improving scoring of particular types of responses—where there is considerable variability in accuracy and response characteristics (e.g., text features, assessment expectations, grade bands) now known to contribute to scoring difficulty—than to rater selection, where there is little variation in accuracy. In sum, the appreciable expense of constructed response scoring^10^ provides considerable opportunity to better leverage this evidence to improve both policy and practice.

Results of the present study further suggest an underappreciation—both in the literature and in practice—of the substantive content of responses as these relate to rater effects. More research is needed to better understand how response content affects the demands of the rating activity and potentially interacts with rater characteristics. Given the considerable influence of individual responses on score quality, further insight could likely inform improvements in score quality in rater-mediated assessment contexts.

A greater understanding of how response content influences scoring accuracy can provide focus for rater training, monitoring, and feedback efforts. Scoring leaders could use knowledge of specific features of responses that introduce scoring difficulty to inform their selection of anchor, qualification, and validity responses. Ensuring that sufficient examples of responses containing these features are represented in the relevant materials will likely improve rater training and monitoring. Additionally, better representing these responses during qualification may increase the predictive validity of the qualification sets. Further, training may be structured to help raters develop awareness of response content likely to increase rating difficulty. By explicitly communicating these features, and the scoring risks they introduce, scoring leaders can help to support raters’ self-regulation of the rating process, in particular so raters are better able to plan, monitor, and regulate their cognition while evaluating written responses. Moreover, such provisions to scoring procedures will minimize barriers to valid score interpretations for the widest possible range of students and likely improve fairness in testing.

Response content information may be used further to inform refinements to scoring rubrics and/or the development of supplemental training materials to afford more reliable scoring. In light of evidence that writing content was categorically more challenging to score than research content, stakeholders might investigate the adoption of item-specific writing rubrics, or, minimally, the development of supplemental material better suited to score atypical responses (which could be identified by their text features).

A priori knowledge of the difficulty of scoring particular response content might further be used to more strategically (1) assign raters to tasks and (2) route responses to raters. By leveraging rater performance data, more accurate raters could be assigned to evaluate response content that is categorically more difficult to score, such as particular assessment expectations or grade bands. Similarly, an automated scoring engine could be used to examine text features of responses and route those responses most likely to be scored inaccurately to specialized raters or scoring leaders.

Finally, results provide the strongest evidence to date of the relative effectiveness of site-based and remote scoring. Note that prior studies (e.g., Knoch et al., 2007) provided site-based but not remote raters with interactive support. In the present study, comparable quality was achieved when providing site-based and remote raters with the same training and level of support. In addition to examining generalizability to other types of items (e.g., mathematics, extended essays), future research should investigate additional affordances of online training and scoring systems to improve rater monitoring and feedback, score quality, and ultimately student classification decisions.

## Data availability statement

The original contributions presented in the study are included in the article/Supplemental material, further inquiries can be directed to the corresponding author.

## Author contributions

The author confirms being the sole contributor of this work and has approved it for publication.

## Conflict of interest

CP is employed by Measurement Incorporated.

## Publisher’s note

All claims expressed in this article are solely those of the authors and do not necessarily represent those of their affiliated organizations, or those of the publisher, the editors and the reviewers. Any product that may be evaluated in this article, or claim that may be made by its manufacturer, is not guaranteed or endorsed by the publisher.

## Author disclaimer

The opinions expressed are those of the author and do not represent the positions or policies of Measurement Incorporated.

## Footnotes

^1^To provide one example, many investigations of rater effects have utilized complete rating designs in which every rater scores every response. In practice, operational scoring programs nearly exclusively use incomplete rating designs, which are far more time and cost effective.

^2^This suggests that the average rater misscored approximately every 7th response by one point relative to the benchmark score.

^3^This variable is calculated at the item level to avoid assuming a practice effect across items within raters.

^4^Mean Decrease in Impurity (MDI), also known as Gini Importance, indicates how often a variable was selected to split a node in a tree-based model and how large the variable’s overall discriminative value was for the classification.

^5^Aligned with the concepts and characteristics the ELA items were designed to measure.

^6^I examine current teaching experience rather than teaching experience as the latter was self-reported and subject to social desirability bias.

^7^The Raftery-Lewis diagnostic (Nhat) provides an estimate of the length of chain required in order to estimate a given posterior quantile (Raftery and Lewis, 1992).

^8^In light of this finding, I omit the remaining interactions from subsequent models.

^9^Such requirements limit the pool of raters and increase the overhead costs of maintaining scoring centers.

^10^Wainer and Thissen (1992) estimate the cost of professional scoring to be greater than five dollars/response (adjusted for inflation), or 3,000 times the cost of machine scoring multiple-choice items.
