# Supplementary material for: Rater characteristics, response content, and scoring contexts: Decomposing the determinates of scoring accuracy
Source: Front Psychol. 2022 Aug 10;13:937097. doi: 10.3389/fpsyg.2022.937097 (PMC9399925; doi:10.3389/fpsyg.2022.937097)
Supplement: Supplementary file 1 [file Data_Sheet_1.pdf]

Appendix A. Checks of Sampling Quality

Appendix Table A1 presents numerical checks of sampling quality for each parameter of Model 6, including the Effective Sample Size (ESS), Monte Carlo Standard Error (MCSE), and Raftery-Lewis diagnostic. The Raftery-Lewis diagnostic (Nhat) provides an estimate of the length of chain required (Nhat) in order to estimate a given posterior quantile (Raftery & Lewis, 1992). Results are provided for both the 2.5% and 97.5% quantiles that form a credible interval, in this case Nhat (quantile) q = (0.025,0.975), assuming (tolerance) r = 0.005 and (probability) s = 0.95. For all parameters, the ESS > 3,000, the MCSE is zero, and the Raftery-Lewis diagnostic is satisfied given that the monitoring chain length of 500,000 exceeds the Nhat values. Overall, results provide strong evidence for convergence and a sufficient sample size.

Appendix Figure A1 shows an example of the visual checks conducted during the MCMC analysis for each parameter estimated. Using the text cohesion variable (β_9) from Model 6 as an example, this figure shows (1) the entire parameter trace (top left), where the “white noise” suggests healthy Gibbs sampling; (2) a kernel density estimate of posterior distribution (top right), which is centered on the mean of 0.027 and appears approximately normal, as expected; (3) the autocorrelation function (middle left), which shows an auto-correlation of approximately 0.45 at a lag of 1, indicating that the next value is somewhat dependent on the previous; (4) the partial autocorrelation function (middle right), which shows minimal correlation after a lag of 1 suggesting there is sufficient independent information in the trace; and finally (5) a plot of estimated MCSE against the number of iterations, which confirms that lengthening the monitoring chains beyond 500,000 would provide little additional precision. For further information about these MCMC diagnostics see Browne, 2019; Jones and Subramanian, 2019.

Appendix A References

Browne, W. J. (2019). *MCMC estimation in MLwiN v3.03*. Centre for Multilevel Modelling, University of Bristol. http://www.bristol.ac.uk/cmm/media/software/mlwin/downloads/manuals/3-03/mcmc-web.pdf

Jones, K., & Subramanian, S. V. (2019). *Developing multilevel models for analysing contextuality, heterogeneity and change using MLwiN 3, Volume 1*. Bristol: University of Bristol, Centre for Multilevel Modelling.

Raftery, A.E. & Lewis, S.M. (1992). How many iterations in the Gibbs sampler? In J.M. Bernado et al. (eds), *Bayesian Statistics 4* (pp. 765–766). Oxford: Oxford University Press.

Appendix Table A1

Numerical Checks of Sampling Quality for Model 6

Parameter	Model 6						
	M	*SD*	Median	95% CrI	ESS	MCSE	Raftery-Lewis diagnostic (Nhat)
Fixed effects							
Intercept (β_0)	0.125	0.008	0.125	0.110, 0.141	3280	0.00	126280, 133320
Time (β_1)	<−.001	<.001	<−.001	−0.000, 0.000	21061	0.00	46040, 45200
Response Content							
Lexical diversity (β_2)	−0.267	0.013	−0.267	−0.293, −0.241	17099	0.00	52360, 51750
Syntactic variety (β_3)	0.641	0.125	0.641	0.394, 0.885	17750	0.00	51140, 50460
Lexical soph. (β_4)	0.758	0.187	0.759	0.391, 1.123	18569	0.00	50970, 51140
Cap. accuracy (β_5)	−0.444	0.119	−0.443	−0.677, −0.209	15523	0.00	53900, 54570
Semantic accuracy (β_6)	−0.648	0.082	−0.647	−0.807, −0.488	15719	0.00	55760, 53250
Temporal con. use (β_7)	0.081	0.089	0.081	−0.094, 0.255	19483	0.00	49370, 48070
Semantic precision (β_8)	−0.206	0.027	−0.206	−0.259, −0.152	18365	0.00	51340, 48480
Text cohesion (β_9)	0.027	0.003	0.027	0.021, 0.032	19564	0.00	48880, 49960
Research expectation	Ref						
Writing expectation (β_{10})	0.044	0.011	0.045	0.026, 0.063	3048	0.00	143200, 148250
Grade band 3–5 (β_{11})	0.025	0.008	0.025	0.008, 0.041	3581	0.00	141690, 132370
Grade band 6–8	Ref						
Grade band 11 (β_{12})	−0.004	0.010	−0.004	−0.023, 0.015	5415	0.00	94950, 100340
Temporal con. use*Writing exp. (β_{13})	0.340	0.100	0.340	0.142, 0.537	32703	0.00	40540, 40610
Semantic precision*Writing exp. (β_{14})	0.190	0.032	0.190	0.129, 0.252	35585	0.00	39480, 39870
Text cohesion*Writing exp. (β_{15})	−0.024	0.003	−0.024	−0.031, −0.018	36242	0.00	39680, 39290
Qual percent exact (β_{16})	<−.001	<.001	<−.001	−0.001, −0.000	30109	0.00	41560, 40950
Qual non-adjacent (β_{17})	0.020	0.004	0.020	0.013, 0.028	42180	0.00	38710, 39100

New/inexperienced	Ref						
Experienced (β_{18})	−0.007	0.007	−0.007	−0.022, 0.007	11629	0.00	63490, 64850
Senior (β_{19})	−0.011	0.008	−0.011	−0.027, 0.005	12316	0.00	61300, 62830
Undergraduate degree	Ref						
Graduate degree (β_{20})	0.004	0.004	0.004	−0.003, 0.012	13036	0.00	60870, 58400
Terminal degree (β_{21})	0.014	0.010	0.014	−0.006, 0.034	13110	0.00	61090, 60870
Current teacher (β_{22})	−0.013	0.008	−0.013	−0.029, 0.003	12261	0.00	64160, 63050
Scoring Context							
Site based (β_{23})	0.008	0.005	0.008	−0.002, 0.019	12700	0.00	61190, 62600
Evening shift (β_{24})	0.012	0.006	0.012	0.001, 0.023	7227	0.00	81030, 88210
Random effects							
Team variance ($\sigma^2_{u0(5)}$)	0.001	<.001	0.001	0.000, 0.001	11095	0.00	56720, 46890
Item variance ($\sigma^2_{u0(4)}$)	0.004	<.001	0.004	0.003, 0.005	22854	0.00	41220, 39680
Rater variance ($\sigma^2_{u0(3)}$)	0.004	<.001	0.004	0.003, 0.004	42162	0.00	38080, 37960
Rater time slope covariance ($\sigma^2_{u01(3)}$)	<.001	<.001	<.001	<.001, <.001	39775	0.00	38330, 38150
Response variance ($\sigma^2_{u0(2)}$)	0.027	<.001	0.027	0.027, 0.028	47113	0.00	37590, 37590
Residual variance (σ^2_{e})	0.100	<.001	0.100	0.100, 0.100	49980	0.00	37340, 37220

Note. ESS = effective sample size, MCSE = Monte Carlo Standard Error, Nhat *q* = (0.025,0.975), *r* = 0.005 and *s* = 0.95

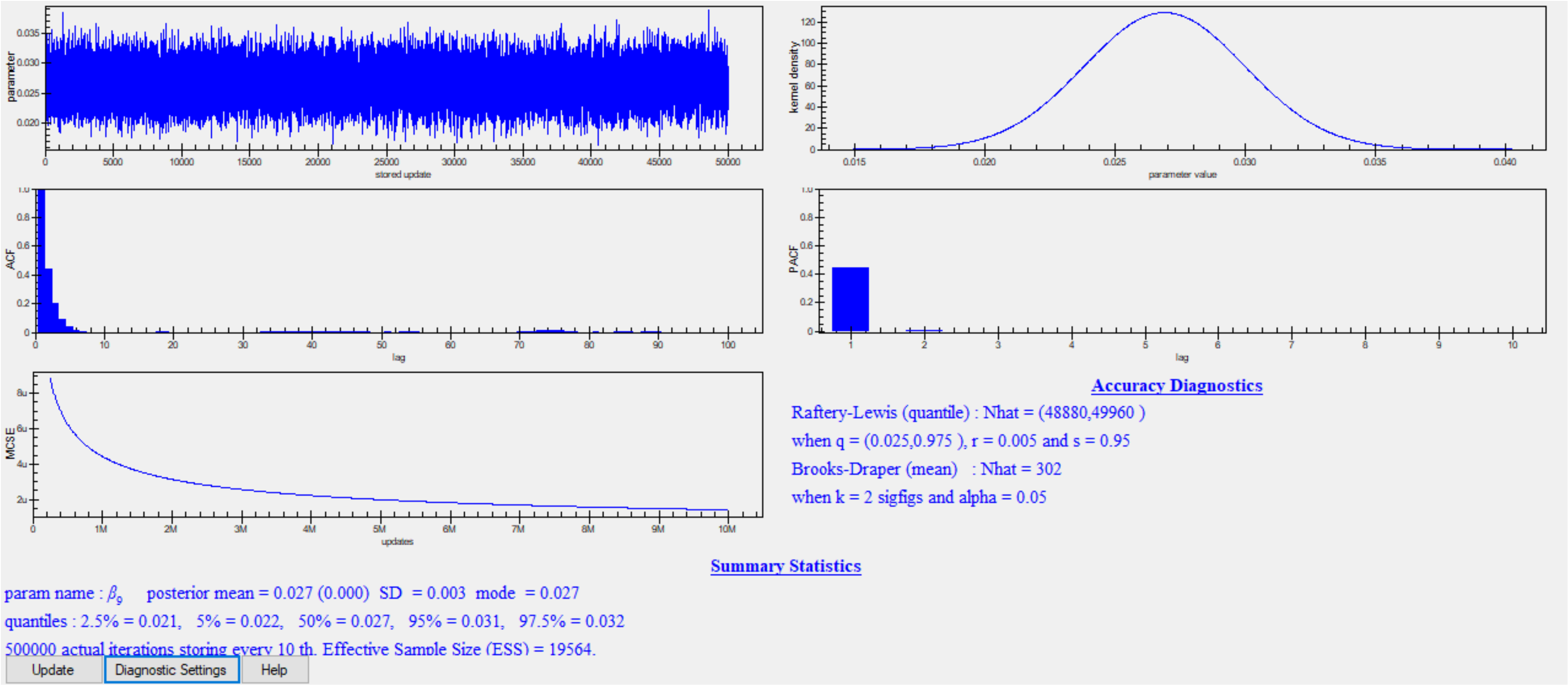

Appendix Figure A1. Example of visual checks conducted during MCMC analysis.

Appendix B. Sensitivity Analysis

Assessment Expectation Classification

In the analyses, three research and nine writing expectations were collapsed into a single category for each, using a dichotomous variable to distinguish between research (0) and writing (1) expectations. In a sensitivity analysis, I examined the potential loss of information from this approach. Recall that the three research expectations differentiated among analysis and integration of information, evaluation of information, and use of evidence. Of the nine writing expectations, each called for writing or revising a short text's (1) introduction, (2) conclusion, or (3) elaboration for one of three purposes: opinion/argumentative, narrative, or informational/explanatory. Here, I re-specify Model 3 as Model 3A, including individual fixed effects for each assessment expectation. As specified, Model 3A allows for a comparison of a reference expectation (research expectation A, requiring analysis and integration of information) with the other 11 expectations. Appendix Table B2 presents results of Model 3A. Results show significant variability in absolute score differences across expectations. In line with results of Model 3, responses associated with writing expectations were generally more difficult to score than responses associated with research expectations. Seven of the nine writing expectations were positively associated with the dependent variable, and the predicted absolute score differences for three of these (expectations F, I, and K) were significantly larger than the value associated with the reference expectation. Compared to Model 2, Model 3A (ΔDIC = −112.2) did not provide better fit than the more parsimonious Model 3 (ΔDIC = −116.3). Therefore, I do not pursue the individual-expectation specification approach in subsequent models.

Appendix Table B2

Parameter Estimates for Model 3A

Parameter	*M*	*SD*	95% CrI
Fixed effects			
Intercept (β_0)	0.152	0.009	0.135, 0.169
Time (β_1)	<−.001	<.001	−0.000, 0.000
Response Content			
Lexical diversity (β_2)	−0.270	0.013	−0.295, −0.244
Syntactic variety (β_3)	0.660	0.125	0.412, 0.902
Lexical soph. (β_4)	0.762	0.186	0.397, 1.125
Cap. accuracy (β_5)	−0.459	0.120	−0.692, −0.226
Semantic accuracy (β_6)	−0.649	0.082	−0.808, −0.490
Temporal con. use (β_7)	0.229	0.079	0.075, 0.383
Semantic precision (β_8)	−0.157	0.026	−0.207, −0.107
Text cohesion (β_9)	0.022	0.003	0.017, 0.027
RS expectation A	Ref		
RS expectation B (β_{10})	−0.031	0.011	−0.052, −0.010
RS expectation C (β_{11})	−0.029	0.010	−0.049, −0.009
WR expectation D (β_{12})	0.014	0.016	−0.017, 0.046
WR expectation E (β_{13})	0.023	0.018	−0.012, 0.058
WR expectation F (β_{14})	0.130	0.022	0.088, 0.173
WR expectation G (β_{15})	−0.025	0.017	−0.058, 0.008
WR expectation H (β_{16})	0.001	0.018	−0.034, 0.036
WR expectation I (β_{17})	0.069	0.021	0.028, 0.111
WR expectation J (β_{18})	−0.027	0.016	−0.059, 0.004
WR expectation K (β_{19})	0.101	0.018	0.067, 0.136
WR expectation L (β_{20})	0.034	0.020	−0.005, 0.072
Grade band 3–5 (β_{21})	0.026	0.008	0.011, 0.042
Grade band 6–8	Ref		
Grade band 11 (β_{22})	−0.003	0.009	−0.021, 0.015
Random effects			
Team variance ($\sigma^2_{u0(5)}$)	0.001	<.001	0.000, 0.001
Item variance ($\sigma^2_{u0(4)}$)	0.003	<.001	0.003, 0.004
Rater variance ($\sigma^2_{u0(3)}$)	0.004	<.001	0.003, 0.004
Rater time slope covariance ($\sigma^2_{u01(3)}$)	<.001	<.001	<.001, <.001
Response variance ($\sigma^2_{u0(2)}$)	0.027	<.001	0.027, 0.028
Residual variance (σ^2_e)	0.100	<.001	0.100, 0.100
DIC	359889.7		

DIC change	−109.5

Note. M = posterior mean, SD = posterior standard deviation, CrI = credible interval of the posterior density estimate.
